# Classification of high-throughput phenotyping data for differentiation among nutrient deficiency in common bean

**DOI:** 10.3389/fpls.2022.931877

**Published:** 2022-07-22

**Authors:** Boris Lazarević, Klaudija Carović-Stanko, Marek Živčak, Dominik Vodnik, Tomislav Javornik, Toni Safner

**Affiliations:** ^1^Department of Plant Nutrition, Faculty of Agriculture, University of Zagreb, Zagreb, Croatia; ^2^Centre of Excellence for Biodiversity and Molecular Plant Breeding, Zagreb, Croatia; ^3^Department of Seed Science and Technology, Faculty of Agriculture Zagreb, University of Zagreb, Zagreb, Croatia; ^4^Institute of Plant and Environmental Sciences, Slovak University of Agriculture, Nitra, Slovakia; ^5^Department of Agronomy, Biotechnical Faculty, University of Ljubljana, Ljubljana, Slovenia; ^6^Department of Plant Breeding, Genetics and Biometrics, Faculty of Agriculture, University of Zagreb, Zagreb, Croatia

**Keywords:** chlorophyll fluorescence imaging, multispectral imaging, 3D multispectral scanning, discriminant analysis, recursive partitioning, nutrient deficiency

## Abstract

The development of automated, image-based, high-throughput plant phenotyping enabled the simultaneous measurement of many plant traits. Big and complex phenotypic datasets require advanced statistical methods which enable the extraction of the most valuable traits when combined with other measurements, interpretation, and understanding of their (eco)physiological background. Nutrient deficiency in plants causes specific symptoms that can be easily detected by multispectral imaging, 3D scanning, and chlorophyll fluorescence measurements. Screening of numerous image-based phenotypic traits of common bean plants grown in nutrient-deficient solutions was conducted to optimize phenotyping and select the most valuable phenotypic traits related to the specific nutrient deficit. Discriminant analysis was used to compare the efficiency of groups of traits obtained by high-throughput phenotyping techniques (chlorophyll fluorescence, multispectral traits, and morphological traits) in discrimination between nutrients [nitrogen (N), phosphorus (P), potassium (K), magnesium (Mg), and iron (Fe)] at early and prolonged deficiency. Furthermore, a recursive partitioning analysis was used to select variables within each group of traits that show the highest accuracy for assigning plants to the respective nutrient deficit treatment. Using the entire set of measured traits, the highest classification success by discriminant function was achieved using multispectral traits. In the subsequent measurements, chlorophyll fluorescence and multispectral traits achieved comparably high classification success. Recursive partitioning analysis was able to intrinsically identify variables within each group of traits and their threshold values that best separate the observations from different nutrient deficiency groups. Again, the highest success in assigning plants into their respective groups was achieved based on selected multispectral traits. Selected chlorophyll fluorescence traits also showed high accuracy for assigning plants into control, Fe, Mg, and P deficit but could not correctly assign K and N deficit plants. This study has shown the usefulness of combining high-throughput phenotyping techniques with advanced data analysis to determine and differentiate nutrient deficiency stress.

## Introduction

Feeding a world population of almost 10 billion people in 2,050 would require raising overall food production by more than 54% from the base year of 2012 (FAO, 2018). To obtain stable and high yields, crop production relies on the application of significant amounts of plant nutrients, especially nitrogen (N), phosphorus (P), and potassium (K) (Stewart et al., 2005). However, modern agriculture faces several challenges that will affect current fertilization practices and other management practices. Through the changes in land use and utilization of different management practices, agriculture significantly contributes to the global greenhouse gas emissions causing climate change (Lynch et al., 2021). In contrast, global climate change will increase the occurrence and intensity of unfavorable environmental conditions subjecting crops to various abiotic stresses (IPCC, 2014), degrading arable soils, and reducing crop nutrient acquisition and utilization from soils (St.Clair and Lynch, 2010). Thus, future agriculture management practices would need to increase productivity, especially on less fertile croplands, increase resource efficiency, and simultaneously decrease its effect on climate by reducing inputs such as fuel, pesticides, and fertilizers (FAO, 2018). The above-mentioned unfavorable conditions in crop production will increase the frequency and severity of plant nutrient deficiencies. Because of their involvement in key physiological processes within the plant, inadequate nutrient supply causes specific nutrient deficiency symptoms (Marschner, 1995). Since each nutrient is included in several physiological and developmental processes, most deficiency symptoms are multisystemic, and it is often hard to connect the visible symptoms with the deficiency of the specific nutrient. The misclassifications among different nutrient deficiencies are frequent. Therefore, there is a need for accurate, early, objective, and non-destructive identification of nutrient deficiency symptoms. For this purpose, modern non-destructive phenotyping techniques combined with advanced data analytics could be used (Zhao et al., 2019; Yang et al., 2020; Singh et al., 2021). Both the United Nations Framework Convention on Climate Change (UNFCCC) and the Fifth Assessment Report of Working Group Two of the Intergovernmental Panel on Climate Change (IPCC WGII AR5) had highlighted technology as a critical resource for ensuring effective adaptation of agriculture to the approaching constraints (UNFCCC, 2013; IPCC, 2014).

Due to the integration of new technologies from computer sciences, engineering, and math sciences with agronomy and life sciences, plant phenotyping evolved from traditional low-throughput, time-consuming, labor-intensive, and subjective task to one of the most advanced research fields in crop sciences (Furbank et al., 2019; van Eeuwijk et al., 2019; Zhao et al., 2019; Yang et al., 2020; Singh et al., 2021).

Plant phenotyping techniques were previously used for discrimination among different nutrient deficiency symptoms. Pacumbaba and Beyl (2011) used spectral reflectance for the nutrient deficiencies analysis in lettuce (*Lactuca sativa* L.) and reported difficulties in discriminating specific nutrient stresses because of the overlapping spectral signatures of nutrient deficient plants. Several authors reported that spectral reflectance at specific wavelengths could detect nutrients within the leaves (Zhang et al., 2013) and discriminate among nutrient-deficient leaves (Debnath et al., 2021). Chlorophyll fluorescence measurements combined with machine-learning methods were sufficient to discriminate among control (no deficiency) and slight, moderate, and strong iron deficiency in rapeseed (*Brassica napus* L.) leaves (Kalaji et al., 2018) and among different nutrient deficiencies in common bean (*Phaseolus vulgaris* L.) (Aleksandrov, 2022) and sunflower (*Helianthus annuus* L.) (Cadet and Samson, 2011).

Although novel phenotyping platforms combine different imaging techniques (RGB, fluorescent, thermal, multispectral, etc.), previous studies of nutrient deficiencies in plants were often focused on a single technique, making it difficult to compare their effectiveness in detecting nutrient deficiency stress. Moreover, complete phenomics information is the foundation of the research in the “-omics” era (Zhao et al., 2019).

On the other hand, by phenotyping lots of plants for lots of parameters simultaneously, such platforms generate a large amount of data that needs to be processed to extract phenotypic information, shifting the phenotyping bottleneck from collecting images to data analysis (Ubbens et al., 2020). In addition, one of the major impediments in plant phenotyping is how to precisely and efficiently evaluate, understand, and interpret these digital image-based features (Zhao et al., 2019). To address this problem, Furbank et al. (2019) suggest the data reduction approach in which advanced statistical methods are used to map and extract traits of interest. Various statistical or machine learning methods, such as linear discriminant analysis, random forests, and support vector machines, are used for biological-image analysis (Rahaman et al., 2019). Linear discriminant analysis (LDA) (Rao, 1948) assumes linearly independent and normally distributed variables and aims to find their linear combinations that best separate the data into predefined classes. A more robust classification approach is to use classification trees (Breiman et al., 1984) which are built using a process of binary recursive partitioning. In this process, data are iteratively split into partitions that best represent classes, which are then further split until no better split can be made. The result is a set of rules, consisting of selected most efficient variables and their thresholds for classifying the data into predefined categories. These rules are less complex than discriminant functions, are generated on original variables (unlike LDA which uses synthetic discriminant functions), and can be easily applied to a new set of data.

This study aims (i) to compare groups of traits obtained by high-throughput phenotyping techniques (chlorophyll fluorescence, multispectral traits, and morphological traits) in their efficiency to discriminate among deficiency symptoms of five different plant nutrients [nitrogen (N), phosphorus (P), potassium (K), magnesium (Mg), and iron (Fe)], (ii) to generate rules, based on the variables within each group of traits, for classification of plants with different nutrient deficit treatments at early and prolonged nutrient deficiency stress, and (iii) to compare the efficiency of two different classification methods (LDA and recursive partitioning) for the classification of plants based on the specific nutrient deficiency.

## Materials and methods

### Experimental setup

The experiment was conducted in a growth chamber under 25/22°C, 16/8 h day/night photoperiod, 65% relative air humidity, and 250 μmol m^−2^ s^−1^ of photosynthetic photon flux density (PPFD) provided by NS12 LED lights (Valoya Oy, Helsinki Finland). Common bean seeds (*Phaseolus vulgaris* L. cv. Ferguson) were planted in two germination trays (containers) with 84 cells (168 in total) and filed with 44 mL/cell of quartz sand. Nine days after planting, 60 equally developed plants were selected, sand was washed from their roots, and they were transferred to six plastic hydroponic trays (60 × 40 × 32 cm) filled with 30 L constantly aerated $\frac{1}{2}$ strength modified Hoagland's solution (Hoagland and Arnon, 1950). Before applying the treatments, plants (10 plants per tray) were left to recover from transplantation for 3 days. Treatments were applied when plants fully developed their first true leaf (unifoliolate). Treatments were represented as nutrient solutions from which different nutrients [nitrogen (N), phosphorus (P), potassium (K), magnesium (Mg), and iron (Fe)] were omitted. These nutrients were selected as the main representatives of five different nutrient groups based on their physiological role in plants (Mengel and Kirkby, 2001). Deficiencies of these nutrients were chosen as treatments since they cause strong effects on plant morphology (Jacob and Lawlor, 1991; Gerardeaux et al., 2010; Rout and Sahoo, 2015; Guo et al., 2016; Mu et al., 2018) as well as metabolic processes, such as photosynthesis, protein and pigment synthesis, osmoregulation, cell wall, and membrane stability (Richter and Rao, 2005; Kalaji et al., 2018; Mu and Chen, 2021; Aleksandrov, 2022). Such effects can be quantified by image-based plant phenotyping. Chemicals used to prepare nutrient solutions are shown in Table 1, whereas the final composition of all treatment nutrient solutions is shown in Table 2. Four measurements (MT1-MT4), every 3 days, were taken during 12 days of growth in treatment solutions. Nutrient solutions were replenished at each measurement time.

**Table 1:** Chemicals used for the preparation of the stock solution and volume of the stock solutions used to produce the treatment solutions.

**Source**	**Molecular weight** **(g mol**^−1^**)**	**Stock** **(mol L**^−1^)*	**Volume added to the final (treatment) solutions** **(mL L**^−1^**)**					
			**Control**	**N**	**P**	**K**	**Mg**	**Fe**
Ca(NO_3_)_2_ ×4H_2_O	236.16	1	2.5	-	2.5	2.5	2.5	2.5
NH_4_NO_3_	80.04	1	1	-	1	1	1	1
K_2_SO_4_	174.26	0.5	2	2	2.5	-	2	2
KH_2_PO_4_	136.09	1	1	1	-	-	1	1
MgSO_4_ ×7H_2_O	246.48	1	1	1	1	1	-	1
Fe-citrate	244.94	0.01	5	5	5	5	5	-
CaCl_2_	110.98	1	0.1	2.5	0.1	0.1	0.1	0.1
NH_4_H_2_PO_4_	115.03	1	-	-	-	1	-	-
H_3_BO_3_	61.83	46.3*	1	1	1	1	1	1
ZnSO_4_ ×7H_2_O	287.56	0.76*	1	1	1	1	1	1
CuSO_4_ ×5H_2_O	249.69	0.32*	1	1	1	1	1	1
MnSO_4_ × H_2_O	169.02	6.51*	1	1	1	1	1	1
H_2_MoO_4_	161.95	0.12*	1	1	1	1	1	1

^*^Micronutrients were produced as a single stock solution, concentrations expressed in mmol L^−1^.

**Table 2:** The concentration of plant nutrients in treatment solutions.

**Plant nutrient**	**The concentration of nutrients in final (treatment) solutions** **(mg L**^−1^**)**					
	**Control**	**N**	**P**	**K**	**Mg**	**Fe**
N	98.0	0.00	98.0	112.0	98.0	98.0
P	31.0	31.0	0.00	31.0	31.0	31.0
K	117.3	117.3	97.8	0.00	117.3	117.3
Ca	104.3	100.3	104.3	104.3	104.3	104.3
Mg	24.3	24.3	24.3	24.3	0.00	24.3
S	64.2	64.2	72.2	32.2	32.2	64.2
Fe	2.85	2.85	2.85	2.85	2.85	0.00
Cl	7.09	177.3	7.09	7.09	7.09	7.09
B	0.50	0.50	0.50	0.50	0.50	0.50
Zn	0.05	0.05	0.05	0.05	0.05	0.05
Cu	0.02	0.02	0.02	0.02	0.02	0.02
Mn	0.36	0.36	0.36	0.36	0.36	0.36
Mo	0.01	0.01	0.01	0.01	0.01	0.01

### Phenotyping measurements

#### Chlorophyll fluorescence imaging

**Figure 1:**
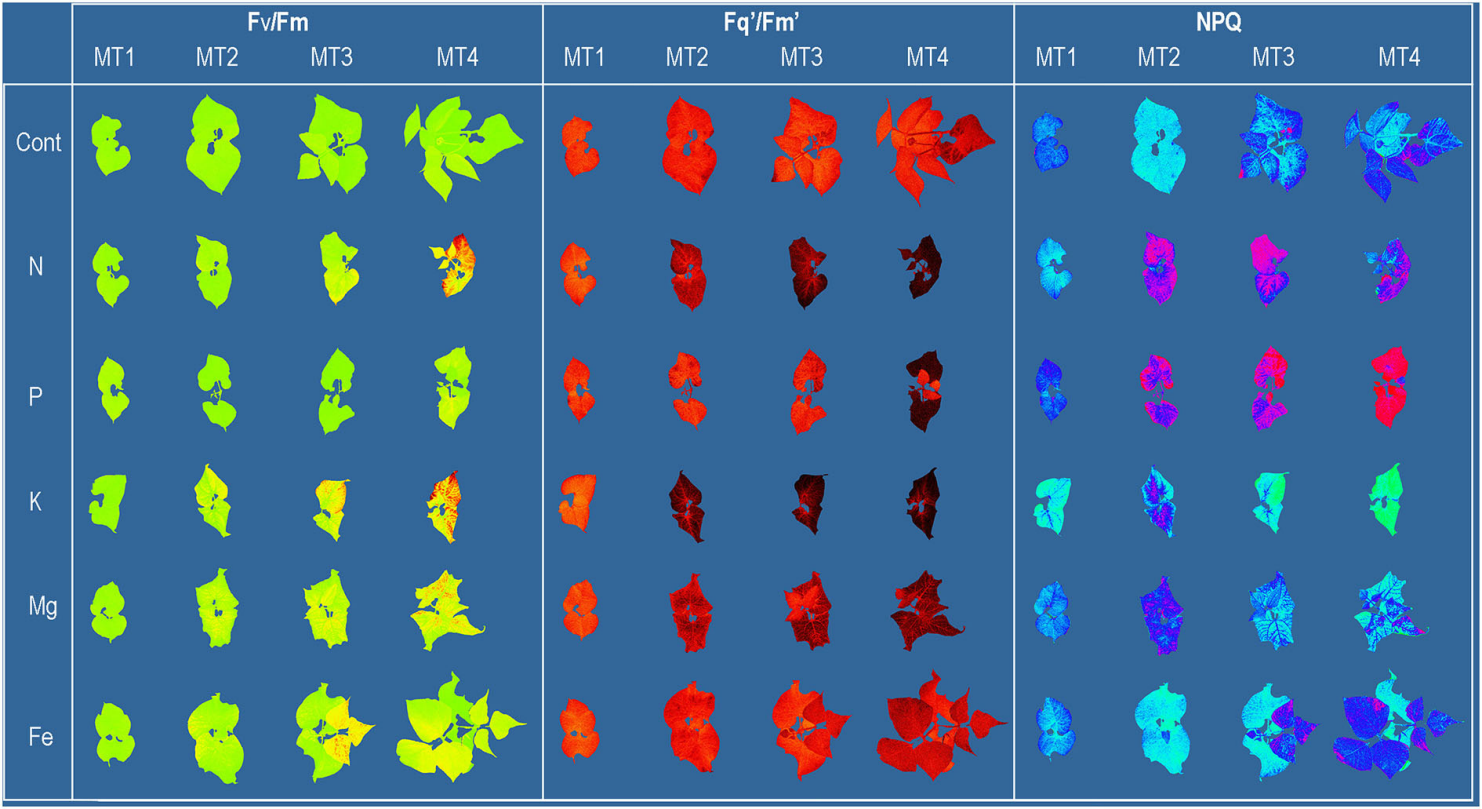
Common bean color and pseudo-color images of maximum quantum yield of PSII (F_v_/F_m_), the effective quantum yield of PSII (F_q_'/F_m_'), and non-photochemical quenching (NPQ) captured by CropReporter at four measurements (MT1-MT4), every 3 days during 12 days of growth in Control [$\frac{1}{2}$ modified Hoagland's solution (Cont)] and solutions without nitrogen (N), phosphorus (P), potassium (K), magnesium (Mg), and iron (Fe).

The whole plant chlorophyll fluorescence imaging was performed using the CropReporter™ (PhenoVation B.V., Wageningen, The Netherlands). Plants were imaged from 70 cm distance. The measurements followed the protocol described in Lazarević et al. (2021) and are briefly described here. Before measurements, plants were dark-adapted for 30 min. For the excitation of photosynthesis, 4,000 μmol m^−2^ s^−1^ red LED light was used. The integration time for capturing the chlorophyll fluorescence image was 200 μs. The minimum chlorophyll fluorescence (F_0_) and maximum chlorophyll fluorescence (F_m_) images were captured after 10 μs and 800 ms, respectively. After the measurement, leaves were relaxed in the dark for 15 s and then adapted to the light using actinic light of 250 μmol m^−2^ s^−1^ for 5 min. Steady-state fluorescence yield (F_s_') was measured before the onset of the saturating pulse, and maximum chlorophyll fluorescence (F${}_{\text{m}}^{{}^{\text{′}}}$) of light-adapted leaves was measured at saturation, using the saturating pulse intensity (4,000 μmol m^−2^ s^−1^). After the measurement, actinic light was turned off, and in the presence of far-red light, minimal fluorescence yield of the illuminated plant (F${}_{0}^{{}^{\text{′}}}$) was estimated. All measured and calculated chlorophyll fluorescence parameters are shown in Table 3. Images of selected chlorophyll fluorescence parameters are shown in Figure 1.

**Table 3:** List of analyzed chlorophyll fluorescence traits (CFT) with abbreviations, equation for calculation, and the reference if appropriate.

**Abbrev**	**Trait**	**Wavelength/equation**
F_0_	Minimum fluorescence of dark-adapted leaves	See description in Material and methods Section
F_m_	Maximum fluorescence of dark-adapted leaves	See description in Material and methods Section
F_s_'	Steady-state fluorescence yield	See description in Material and methods Section
F_m_'	Maximum chlorophyll fluorescence of light-adapted leaves	See description in Material and methods Section
F_o_'	Minimum fluorescence yield of illuminated plant	See description in Material and methods Section
F_v_/F_m_	Maximum efficiency of photosystem two	F_v_/F_m_ = (F_m_-F_0_)/F_m_ (Kitajima and Butler, 1975)
F_q_'/F_m_'	Effective quantum yield of photosystem two	F_q_'/F_m_' = (F_m_' - F_s_')/F_m_' (Genty et al., 1989)
ETR	Electron transport rate	ETR = F_q_'/F_m_' × PPFD × (0.5) (Genty et al., 1989)
NPQ	Non-photochemical quenching	NPQ = (F_m_ - F_m_')/F_m_' (Bilger and Björkman, 1990)
qP	Coefficient of photochemical quenching	qP = (F_m_' - F_s_')/F_v_ (Schreiber et al., 1986)
qN	Coefficient of non-photochemical quenching	qN = 1 – (F_m_' – F_o_')/(F_m_ – F_o_) (Schreiber et al., 1986)
qL	Estimation of “open” reaction centers on basis of a lake model	qL = ((F_m_' - F_s_') × F_o_'))/((F_m_' - F_o_') × F_s_')) (Kramer et al., 2004)
(Φnq)	Quantum yield of non-regulated non-photochemical energy loss in PSII	Φnq = 1/(NPQ + 1 + qL(F_m_/F_o_ - 1)) (Genty et al., 1996)
(Φnpq)	Quantum yield of regulated non-photochemical energy loss in PSII	Φnpq = 1 - ΦpsII - 1/(NPQ + 1 + qL(F_m_/F_o_ - 1)) (Genty et al., 1996)

#### Multispectral imaging

After chlorophyll fluorescence measurement, plants were again illuminated with 250 μmol m^−2^ s^−1^ and color, and spectral reflectance images were captured using CropReporter™ (PhenoVation B.V., Wageningen, The Netherlands). Multispectral images were captured at red (R_Red_-640 nm), green (R_Green_ – 550 nm), blue (R_Blue_ – 475 nm), specific green (R_SpcGrn_ – 510–590 nm), chlorophyll reflectance (R_Chl_ – 730 nm), near infra-red (R_NIR_ – 769 nm), and far-red (R_FarRed_ – 710 nm) reflectance. From measured reflectance chlorophyll index (CHI) (Gitelson et al., 2003), anthocyanin index (ARI) (Gitelson et al., 2001), Hue (0–360°), saturation (SAT), and value (VAL) were calculated (Table 4).

**Table 4:** List of analyzed multispectral traits (MST) with abbreviations, wavelength for measurement or equation for calculation, and the reference if appropriate.

**Abbrev**	**Trait**	**Wavelength/equation**
R_Red_, R_Green, _ R_Blue_	Reflectance in Red, Green and Blue	640, 550, and 475 nm
R_SpcGrn, _ R_FarRed, _ R_NIR_	Reflectance in Specific Green, Far Red, Near Infra-Red	510–590 nm, 710 nm, and 769 nm
R_Chl_	Reflectance Specific to Chlorophyll	730 nm
HUE	Hue (0–360°)	HUE = 60 × (0 + (R_Green_ - R_Blue_) / (max-min)), if max = R_Red_; HUE = 60 × (2 + (R_Blue_ - R_Red_) / (max-min)), if max = R_Green_; HUE = 60 × (4 + (R_Red_ - R_Green_) / (max-min)) if max = R_Blue_; 360 was added in case HUE <0
SAT	Saturation (0–1)	SAT = (max – min) / (max + min) if VAL > 0.5, or SAT = (max – min) / (2.0 – max – min) if VAL <0.5, where max and min are selected from the R_Red_, R_Green_, R_Blue_
VAL	Value (0–1)	VAL = (max + min) / 2; where max and min are selected from the R_Red_, R_Green_, R_Blue_
ARI	Anthocyanin Index	ARI = (R_550_)^−1^ - (R_700_)^−1^ (Gitelson et al., 2001)
CHI	Chlorophyll Index	CHI = (R_700_)^−1^ – (R_769_)^−1^ (Gitelson et al., 2003)
NDVI	Normalized Differential Vegetation Index	NDVI = (R_NIR_-R_Red_)/(R_NIR_+R_Red_) (Rouse et al., 1974)
PSRI	Plant Senescence Reflectance Index	PSRI = (R_Red_ – R_Gren_)/(R_NIR_) (Merzlyak et al., 1999)
NPCI	Normalized Pigments Chlorophyll Ratio Index	NPCI = (R_Red_ – R_Blue_)/(R_Red_ + R_Blue_) (Peñuelas et al., 1995)
GLI	Green Leaf Index	GLI = (2 x R_Green_ – R_Red_ – R_Blue_) / (2 x R_Green_ + R_Red_ + R_Blue_) (Gobron et al., 2000)

#### 3D multispectral scanning

**Figure 2:**
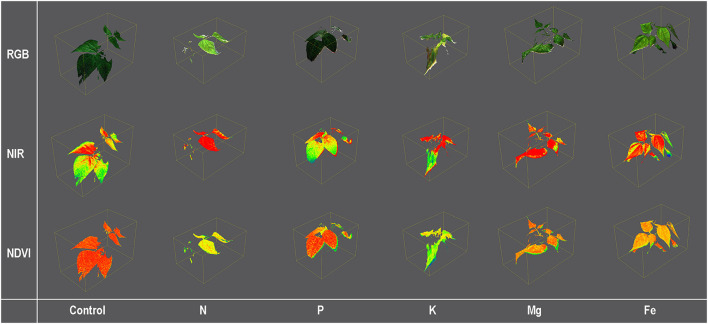
Color [Red, Green, and Blue (RGB)] and pseudo-color [Near Infra-Red (NIR) and Normalized Differential Vegetation Index (NDVI)] images of 3D common bean plants scanned by PlantEye F500 grown for 9 days (MT3) in treatment solutions [$\frac{1}{2}$ modified Hoagland's solution (Control), and solutions without nitrogen (N), phosphorus (P), potassium (K), magnesium (Mg), and iron (Fe)].

Plants were scanned from a 70-cm distance using the PlantEye F500 multispectral 3D scanner (Phenospex, Heerlen, The Netherlands) in red (peak wavelength 620–645 nm), green (peak wavelength 530–540 nm), blue (peak wavelength 460–485 nm), near-infrared (peak wavelength 820–850 nm), and the 3D laser (940 nm). A detailed description of the PlantEye, the scanning resolution, and the reconstruction of the 3D plant model are given in Lazarević et al. (2021). From the 3D plant model, different morphological parameters and vegetation indices were calculated using the HortControl software (Phenospex, Heerlen, The Netherlands). Examples of 3D plant models with some analyzed traits are given in Figure 2.

Calculated vegetation indices are Green leaf index (GLI), Normalized Differential Vegetation Index (NDVI) (Rouse et al., 1974), Plant Senescence Reflectance Index (PSRI) (Merzlyak et al., 1999), and Normalized Pigments Chlorophyll Ratio Index (NPCI) (Peñuelas et al., 1995; Table 4).

Calculation of morphological parameters starts from the 3D point cloud from which the 3D plant model is built. All points that belong to the same sector are triangulated. To create a triangle in the point cloud, the points should be close to each other with no other point in between (Y-resolution 1 mm, X- resolution 0.19 mm, and Z-resolution <0.1 mm). Calculated morphological parameters are plant Height (H; mm), Leaf area projected (LAP; mm^2^), Total leaf area (TLA; mm^2^), Digital volume (DV; mm^3^), Leaf area index (LAI, mm^2^ mm^−2^), Leaf inclination (LINC; mm^2^ mm^−2^), Leaf angle (LANG; °), and Light penetration depth (LPD; mm). The list of all measured morphological traits, abbreviations, and equations is given in Table 5.

**Table 5:** List of analyzed morphological (MORPH) with abbreviations, equations, or descriptions for calculation.

**Abbrev**	**Trait**	**Wavelength/equation**
H	Plant Height (mm)	Calculated as distribution of elementary triangles along the z-axis
DV	Digital volume (mm^3^)	Calculated as the product of height and 3D leaf area
LAP	Leaf area projected (mm^2^)	Calculated as an area of the projection of all elementary triangles on X-Y plan
TLA	Total Leaf Area (mm^2^)	Calculated as sum of all triangle domains, where each domain represents group of triangles that forms a uniform surface
LAI	Leaf area index (mm^2^ mm^−2^)	Calculated as TLA/sector size
LINC	Leaf Inclination (mm^2^ mm^−2^)	Describes how leaves on plant are erected and calculated as TLA/LAP
LANG	Leaf angle (°)	
LPD	Light penetration depth, mm)	Measured by the deepest point in which the laser can penetrate the canopy along the z-axis

#### Mineral content analysis

Mineral content analysis was performed in the shoots from the experimental plants, harvested at the end of the experiment (12 days after the onset of the treatments). Plant samples were dried at 70°C until constant weight using VL 180 Prime drying oven (VWR International, Leuven, Germany). Dried samples were ground using M 20 Universal mill (IKA®-Werke GmbH & Co, Germany). To obtain enough dry samples for the mineral content analysis, three composited samples were created by combining material obtained from 10 plants from each nutrient deficiency treatment. Total N was determined using the Modified Kjeldahl method (HRN ISO 11261:2004). After the digestion of plant material in a microwave oven (Milestone Ethos Up, Milestone Srl, Italy) using the mixture of nitric acid (HNO_3_) and perchloric acid (HClO_4_), K was determined using a flame photometer (PFP-7, Jenway, UK) and P using a spectrophotometer (Evolution 60 S, Thermo Fisher Scientific, Finland), whereas concentrations of Mg and Fe were determined using an atomic absorption spectrometer (AAS Solar, Thermo Fisher Scientific, Finland).

#### Statistical analysis

Obtained plant phenotypic traits were grouped into three groups: chlorophyll fluorescence traits (CFT), multispectral traits (MST), and morphological traits (MORPH).

We used linear discriminant analysis to estimate the best possible classification between treatments at each measurement time (Fisher, 1936) based on each group of traits. This method projects data from a D dimensional feature space down to a reduced number of dimensions (to a maximum of the number of classes−1 dimension) to maximize the variability between the classes and reduce the variability within the classes. As a result, it finds discriminant functions (linear combinations of all predictor variables) that best separate the data into predefined groups (treatments). The data were then reclassified into treatments according to the values of the discriminant function scores for each data point. The efficiency of classification based on linear discriminant analysis was estimated as the ratio of correctly classified plants and the total number of analyzed plants (60). Discriminant analysis was performed using package MASS (Venables and Ripley, 2002) in R, with the syntax: *lda.model*<*- lda(treatment*~*., data* = *df)*.

Within each group of traits (CFT, MST, and MORPH) at each measurement time (MT), we used recursive partitioning to identify variables and their threshold values that best separate the observations from different treatments (Breiman et al., 1984). This method is used to build classification or regression models using a multi-stage procedure where the resulting models can be represented as decision trees, making them easy to visualize. At each stage, the variable that best separates the data into groups defined by the classification variable (treatment) is selected, and data are subsequently split until the model reaches the best possible classification of data into predefined groups (treatments). The classification tree shows the nodes where the tree is split according to the threshold value of the selected variable and the edges that direct the outcome of the splits to the next node until they reach the leaves that are terminal nodes which show the classification outcome. The efficiency of the selected binary trees was estimated by the ratio of correctly classified data and the total number of data (60). Recursive partitioning was performed using R package rpart (Therneau and Atkinson, 2022), with the syntax: *part.model*<*- rpart(treatment*~*., data* = *df)*.

## Results

### Plant mineral content

Nutrient content in common bean shoots harvested at MT4 is shown in Supplementary Table 1. Nutrient concentrations in plant shoots correspond to the nutrient solution treatments and confirm the specific nutrient deficiency in plants from each treatment. The first visible symptoms were detected at MT2 in N deficiency as pale green leaves and K deficiency as blotching chlorosis and small necrotic spots. The latest visible symptoms development was found in Fe deficiency (at MT3) as chlorosis in young leaves.

### Chlorophyll fluorescence traits

Discriminant analysis showed that classification success for separation among nutrient deficiency treatments (Control, N, P, K, Mg, and Fe) using chlorophyll fluorescence traits (CFT) achieved 80% (48/60 correctly classified plants) at MT1 and 98.3% (59/60 correctly classified plants) for MT2, MT3, and MT4, respectively (Supplementary Table 2).

At MT1, variables belonging to CFT were most successful for the classification of plants from the control group, with the misclassification of only one plant into the Fe treatment group. In contrast, the lowest success was obtained for the K treatment (70% accuracy). At MT2, one plant from N treatment was misclassified as Mg treatment, at MT3, one plant from control treatment was misclassified as Fe treatment, and at MT4, one plant from K treatment was misclassified as Mg treatment group (Supplementary Table 2).

**Figure 3:**
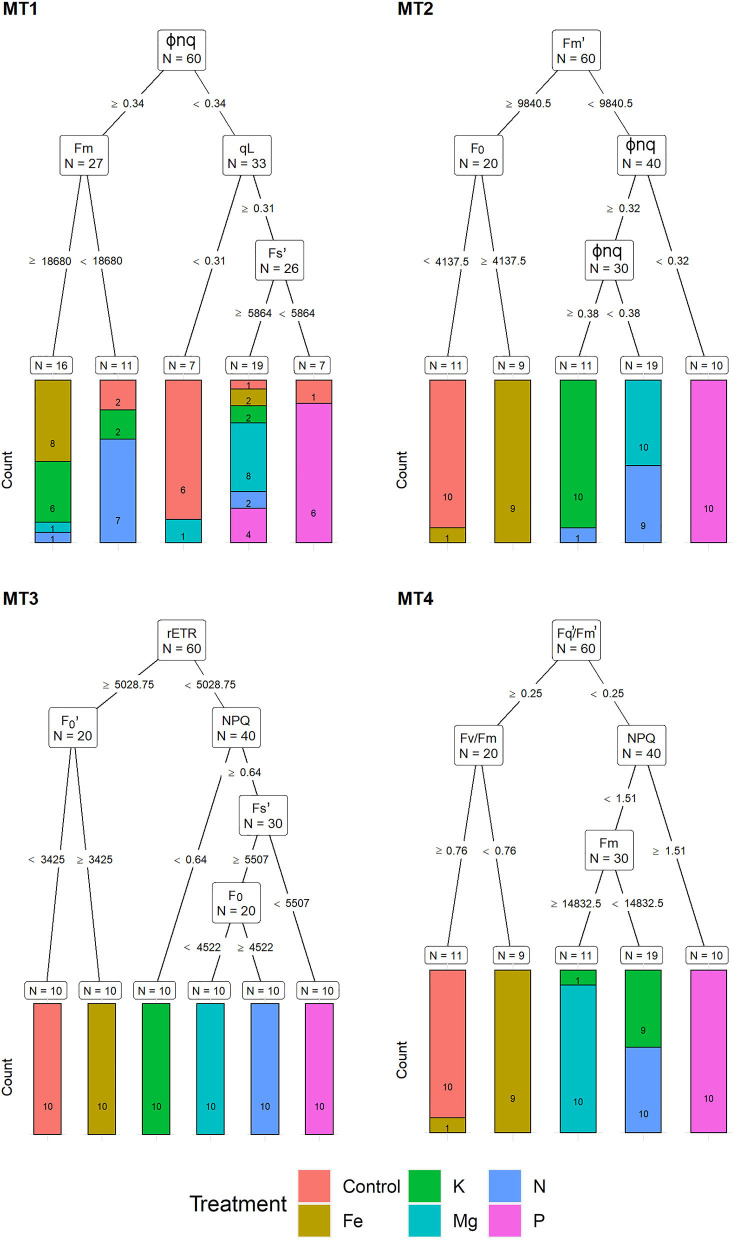
Visualization of classification tree for chlorophyll fluorescence traits (CFT). Each node shows the variable chosen as the best for the split in the data and the number of observations at that node (N). On the edges, between nodes, are threshold values of the split variables. Bar charts at each terminal node (leaf) represent the numbers of observations classified into each treatment (indicated by different colors). MT1 to MT4 represent measurement times.

For the MT1, a recursive partitioning model based on chlorophyll fluorescence traits correctly assigned 58.5% of the plants to their respective treatments. Variables selected by this model were Φnq, F_m_, qL, and F${}_{\text{s}}^{{}^{\text{′}}}$ (Figure 3). This model performed best for assigning Fe and Mg deficiencies (both with 80% accuracy) and could not correctly assign any of the K deficiency plants but instead assigned them as Fe (60%), Mg, and N treatments (20% in each group). The recursive partitioning model for the MT2 included variables F_m_, F_0_ and Φnq, and was able to assign 81.7% of all plants correctly. At MT2, the assignment was 100% accurate for K, Mg, P, and control treatments, while none of the plants from the N treatment could be appropriately assigned and was misclassified as either Mg (90%) or K (10%) (Figure 3). At the MT3, the model included rETR, F${}_{0}^{{}^{\text{′}}}$, NPQ, F${}_{\text{s}}^{{}^{\text{′}}}$, and F_0_ and achieved 100% accurate classification. At MT4, the model included F${}_{\text{q}}^{{}^{\text{′}}}$/F${}_{\text{m}}^{{}^{\text{′}}}$, F_v_/F_m_, NPQ, and F_m_, resulting in 81.7% of correctly assigned plants. Same as for MT1, this model could not correctly assign any of the K plants but instead classified them as N (90%) or Mg (10%) (Figure 3).

### Multispectral traits

The highest classification success based on discriminant functions, when compared among different groups of traits, was achieved by MST. Namely, discriminant analysis based on MST correctly classified 91.6% plants at MT1, 93.3% plants at MT2, 95% plants at MT3, and 100% plants at MT4 into their respective nutrient deficiency groups (Supplementary Table 3). At MT1, using variables from MST discriminant analysis correctly classified plants belonging to the control, N, Mg, and Fe groups, and misclassified three plants belonging to the K deficiency group into Mg, N, and P groups and two plants belonging to the P deficiency group into N deficiency group. At MT2, most misclassifications were found between Fe and control treatment, whereas at MT3, most misclassifications occurred between Fe and Mg treatments (Supplementary Table 3).

**Figure 4:**
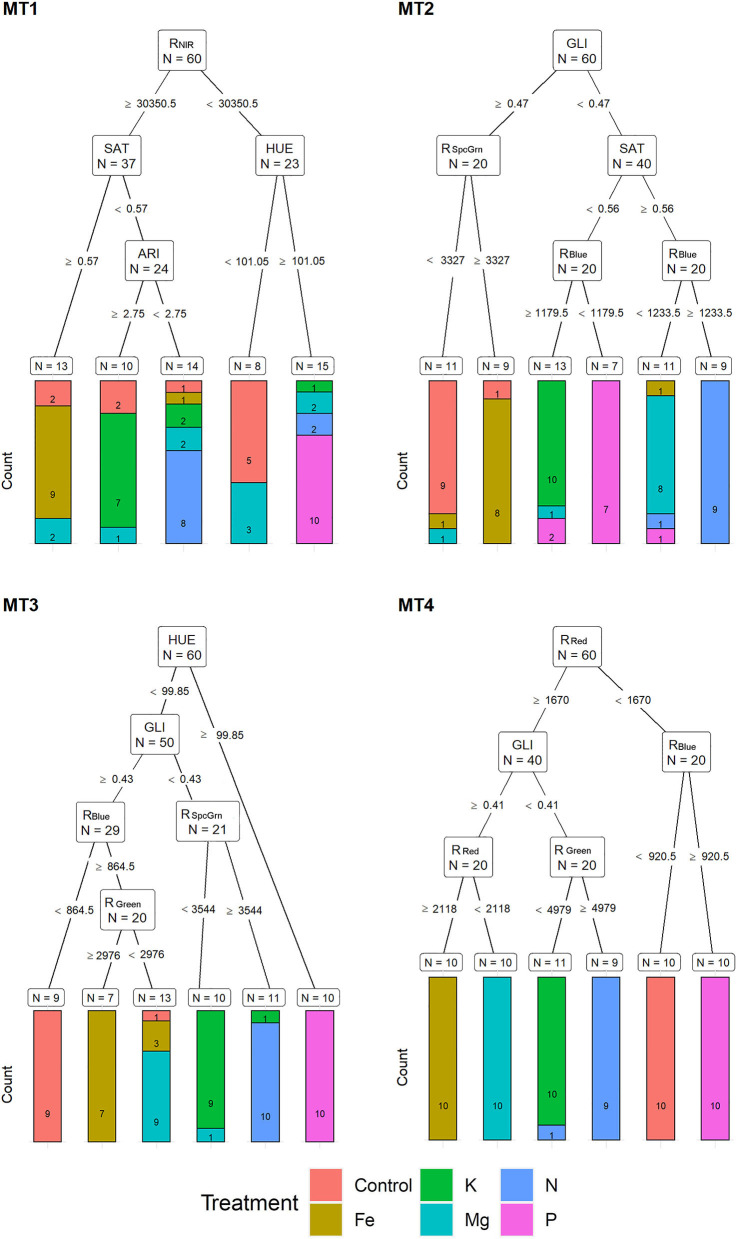
Visualization of classification tree for multispectral traits (MST). Each node shows the variable chosen as the best for the split in the data and the number of observations at that node (N). On the edges, between nodes, are threshold values of the split variables. Bar charts at each terminal node (leaf) represent the numbers of observations classified into each treatment (indicated by different colors). MT1 to MT4 represent measurement times.

Based on MST, for MT1, recursive partitioning model was able to correctly assign 65% of the plants to their respective treatments. Variables selected by this model were R_NIR_, SAT, HUE, and ARI. This model performed best for assigning P (with 100% accuracy), Fe (90% accuracy), and N (80% accuracy) and could not correctly assign any of the Mg deficiency plants (Figure 4). The recursive partitioning model for the MT2 included variables GLI, R_SpcGrn_, SAT, and R_Blue_ and assigned 85% of all plants correctly. At MT2, the assignment was 100% accurate for K, 90% for control and N, 80% for P, and 70% for Mg treatment (Figure 4). At the MT3, the model included HUE, GLI, R_SpcGrn_, R_Blue_, and R_Green_ and achieved 90% accuracy classification. This model performed best for assigning P (with 100% accuracy, already based on HUE), N (10% accuracy), Mg, control, and K (each with 80% accuracy) (Figure 4).

At MT4, the model included R_Red_, R_Blue_, GLI, and R_Green_ and resulted in 98.3% of correctly assigned plants to their respective groups. This model misclassified only 10% of the N treatment plant (assigned to K) (Figure 4).

### Morphological traits

Compared to CFT and MST, discriminant analysis among nutrient deficiency groups based on MORPH achieved the lowest classification success at all measurement times, 58.3% (MT1), 73.3% (MT2), 86.6% (MT3), and 80% (MT4) (Supplementary Table 4). At MT1 highest classification achievement was obtained for N treatment (80%) and lowest for K (30%). However, at latter measurement times, the lowest classification was achieved for plants from the N treatment group (50, 80, and 70%, at MT2, MT3, and MT4, respectively). For other nutrient deficiency groups, classification achievements varied from 70 to 100% (Supplementary Table 4).

**Figure 5:**
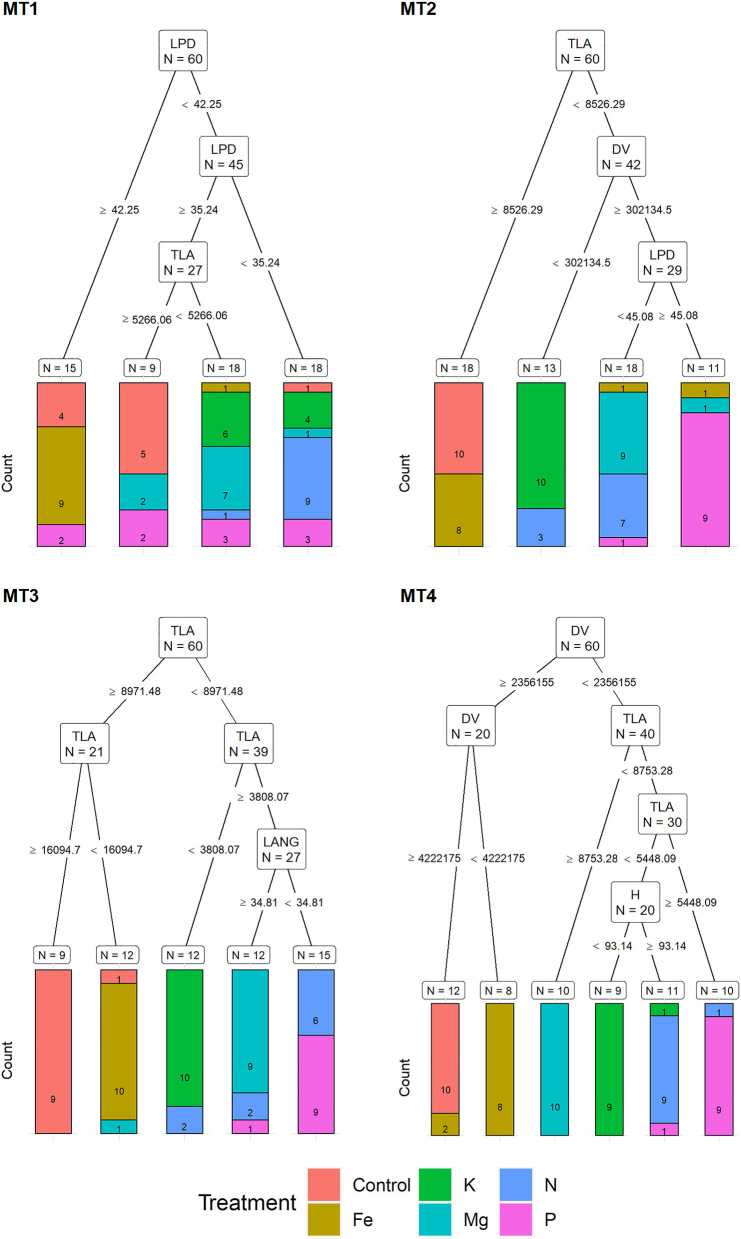
Visualization of classification tree for morphological traits (MORPH). Each node shows the variable chosen as the best for the split in the data and the number of observations at that node (N). On the edges, between nodes, are threshold values of the split variables. Bar charts at each terminal node (leaf) represent the numbers of observations classified into each treatment (indicated by different colors). MT1 to MT4 represent measurement times.

Recursive partitioning models based on MORPH correctly assigned 50% of the plants to their respective treatments at MT1. Variables selected by this model were LPD and TLA. This model performed best for assigning Fe and N treatment (with 90% accuracy) and could not correctly assign any of the K and P deficiency plants (Figure 5).

The recursive partitioning model for the MT2 included variables TLA, DV, and LPD and assigned 63.3% of all plants correctly. At MT2, the assignment was 100% accurate for K and control and 90% for Mg and P treatments, whereas it was not able to correctly assign any of the P and N plants (Figure 5). At the MT3, the model included TLA and LANG and achieved a 78.3% accuracy classification. This model achieved high assignment for Fe and K (both with 100% accuracy) and the control, Mg, and P with 90%. However, it did not be able to assign plants from the N treatment correctly (Figure 5).

At MT4, the model included DV, TLA, and H, resulting in 91.6% of correctly assigned plants to their respective groups. This model misclassified only 10% of plants from N, K, and P treatment groups (Figure 5).

### Comparison of LDA and recursive partitioning

**Figure 6:**
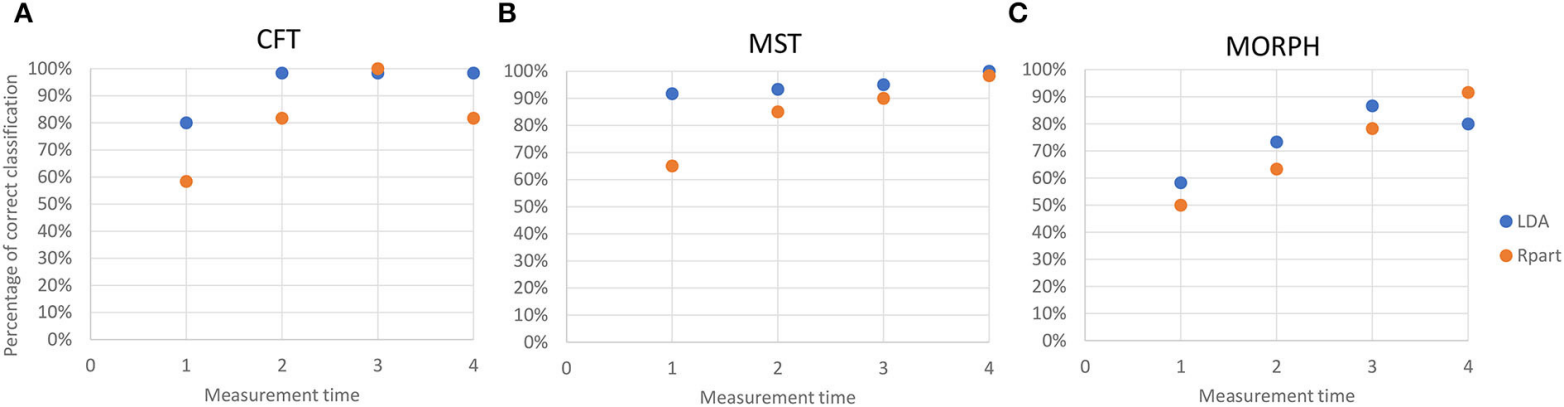
Accuracy of the reclassification of data into correct categories by linear discriminant analysis (blue dots) and recursive partitioning (orange dots) at each measurement time for **(A)** chlorophyll fluorescence traits (CFT); **(B)** multispectral traits (MST); and **(C)** morphological traits (MORPH). Accuracy is estimated as (Number of correctly classified data/Total number of data).

For almost all measurement times and all sets of variables, except MT3 for CFT and MT4 for MORPH, LDA outperformed the recursive partitioning in the percentage of correct classifications (Figure 6). However, the classification trees obtained by recursive partition resulted in more specific sets of classification rules that include only the select number of original variables and give exact classification thresholds for them.

## Discussion

Techniques that combine new digital technologies with advanced data processing represent the most promising scientific tools to overcome modern agricultural production challenges (UNFCCC, 2013; IPCC, 2014). One of such techniques is the high-throughput phenotyping which has been already proven very useful in plant breeding (van Eeuwijk et al., 2019) as well as for studying complex physiological responses of plants to various environmental factors (Munns et al., 2010; Dhondt et al., 2013; Humplik et al., 2015; Wang H. et al., 2018). Nutrient deficiencies are common in agricultural production, and optimal nutrition is necessary for achieving high and stable yields.

This study aimed to compare the efficacy of the traits obtained by advanced plant phenotyping techniques (chlorophyll fluorescence, multispectral imaging, and 3D multispectral scanning) in the discrimination between symptoms of deficiency of five different plant nutrients, nitrogen (N), phosphorus (P), potassium (K), magnesium (Mg), and iron (Fe), and to identify traits that best separate observations from different nutrient deficiency treatments in early and prolonged nutrient deficiency stress.

Compared to reference nutrient content in common bean leaf (Bergmann, 1992; Hochmuth et al., 2018), plant nutrient analysis confirmed that the treatments caused deficiencies of targeted nutrients in the aboveground organs of experimental plants.

At early nutrient deficiency stress (MT1), the highest classification success for separation among nutrient deficiency treatments by discriminant analysis was achieved using multispectral traits (91.6%) whereas the lowest using morphological traits (58.3%). In the subsequent measurements (MT2-MT4), chlorophyll fluorescence and multispectral traits achieved comparable high classification success for separation among nutrient deficiency treatments by discriminant analysis. Also, similar to the discriminant analysis, MT1 recursive partitioning based on selected multispectral traits achieved the highest success in the assignment of plants into their respective groups, whereas at later stages (MT2-MT4), assignment success achieved with chlorophyll fluorescence traits was comparable to those achieved by multispectral traits and were lowest in the case of selected morphological traits. These results indicate that nutrient deficiency has the fastest and most pronounced effect on leaf light absorption/reflection properties. Furthermore, plants with different nutrient deficiencies in the early stages of stress differ most in their reflectance features.

Multispectral traits identified by recursive partitioning show the possibility for effective separation of the observations from different nutrient deficiency treatments. However, different multispectral traits were selected at different measurement times, indicating temporal changes in leaf absorption/reflection properties during stress development. From MT2 to MT4, the most important traits were GLI, R_SpcGrn_, R_Blue_, and R_Green_, while at MT4, recursive partitioning analysis also included the R_Red_.

Multispectral traits selected by recursive partitioning showed high assignment accuracy; however, partial misclassification occurred between Fe and control, N and Mg, and K and P, at an early stage (MT1 and MT2), and between Fe and Mg, K and N, and P and control, in the prolonged stress (MT3 and MT4). Discriminant analysis based on the whole set of multispectral traits showed higher classification success. However, it showed a similar misclassification pattern as described for recursive partitioning. Because multispectral traits are related to leaf pigment content (Blackburn, 2007) and the fact that the highest absorption by chlorophyll molecules occurs in blue and red wavelengths, decreased chlorophyll content in the N, K, Mg, and Fe deficiency at MT4 caused the clear separation of these treatments compared to plants from control and P treatment using only reflection in red (R_Red_). Phosphorus deficiency is known for developing dark green to bluish green leaf color (Marschner, 1995) and was also separated from control by the R_Blue_ (at MT4). Li et al. (2006) also found more difficulties in discrimination among control and P-deficit *Brassica chinensis* leaves using visible reflectance spectrum and explained it by a slight reduction of chlorophyll content under P deficiency treatment compared to control. Moreover, some experiments (Abadia et al., 1987) show increased chlorophyll levels per unit of leaf area in P-deficit sugar beet (*Beta vulgaris*) leaves.

Further, higher similarities in multispectral traits between control and Fe treatment at the early phase (MT1 and MT2) are probably related to lower plant needs for Fe than other macronutrients, and thus the slower symptom development under Fe deficiency. At later stages (MT3 and MT4), similarities in Mg and Fe deficiency are probably related to the impaired chlorophyll synthesis (Rout and Sahoo, 2015), which caused similar absorption/reflection properties of leaves from these two treatments. However, Fe and Mg deficiencies have different patterns of symptom development on a whole plant level (young leaves vs. old leaves) (Figure 1). Thus, better classification could probably be obtained if the signal from the upper and lower leaves could be separated. Prolonged K deficiency includes brown scorching and chlorosis, which is directly related to ROS formation and chlorophyll degradation (Cakmak, 2005) and has a similar effect on light absorption/reflection as chlorosis caused by N deficiency, characterized by increased R_Red_ and decreased GLI at MT4.

Although chlorophyll fluorescence is one of the most frequently used techniques for the determination and quantification of abiotic stress (Maxwell and Johnson, 2000; Murchie and Lawson, 2013), low classification success obtained using chlorophyll fluorescence traits at early nutrient deficiency stress (MT1) can be explained by several short-term stress avoidance mechanisms which provided early protection of photosynthesis from the stress-induced damage (Rungrat et al., 2016). As with the multispectral traits, during measurements (MT1-MT4), recursive partitioning selected different chlorophyll fluorescence traits indicating changes in photoprotective, photochemical, and non-photochemical processes during the development of nutrient deficiency symptoms. In the early stages of stress, best assignment into their respective nutrient deficiency groups was achieved using Φnq, F_m_, qL, and F_s_ at MT1 and F_m_, F_0_, and Φnq at MT2, whereas in the latter stages (MT3 and MT4), recursive partitioning also included rETR, NPQ, F_v_/F_m_, and F_q_'/F_m_'. These results indicate that early nutrient deficiency stresses are more related to the less efficient energy transfer among the antennae complexes toward the PSII reaction centers (F_0_ and F_m_) and with the number of open reaction centers (qL) (Maxwell and Johnson, 2000; Murchie and Lawson, 2013). During the latter stages of stress, selected parameters indicate higher differences in damage of PSII (F_v_/F_m_ and F_q_'/F_m_'), decrease in photosynthetic rate (Fq'/Fm' and rETR), and regulation of energy dissipation (NPQ) (Maxwell and Johnson, 2000; Murchie and Lawson, 2013). Similar to our results, Kalaji et al. (2018) quotes increased F_0_ and F_m_ and decreased quantum yield in nutrient-deficit rapeseed plants, whereas Aleksandrov (2022) found increased F_0_ and F_m_ and decreased rETR and decreased the number of open reaction centers in N, P, and Fe deficit common bean plants. Based on selected chlorophyll fluorescence traits, recursive partitioning could not correctly assign plants from K deficiency treatment at MT1 and MT4 and N treatment at MT2. Aleksandrov (2022), using chlorophyll fluorescence, quotes only slight differences between control and K deficit common bean leaves and stated that K deficiency did not significantly affect the photosynthetic apparatus. There were no misclassifications between plants from control and K deficit treatment in our experiment. Using selected chlorophyll to fluoresce traits, plants from K deficit treatment were assigned to N, Mg, and Fe deficiency groups at MT1 and N and Mg groups at MT4. In addition, at MT2, plants from N deficiency treatment were also assigned to the Mg treatment group. These results indicate similarities in selected chlorophyll fluorescence traits among common bean K, Mg, and N deficiency. However, using the whole set of chlorophyll fluorescence traits in discriminant function, 70 and 90% of classification success were achieved for the K group at MT1 and MT4, respectively, whereas 90% for the N group at MT2, indicating the importance of traits which were omitted by the recursive partitioning for correct assignment of K and N deficit plants. This close relation among N, Mg, and K deficit plants in CFT traits at MT2-MT4 could be explained by their similarities in affecting plant photosynthetic apparatus. For example, Mu and Chen (2021) quoted that N deficiency reduces the content of chlorophyll, photosystems, and light-harvesting centers, and thus reduces light absorption and photochemistry and increases NPQ. In response to Mg and K deficiency, plants protect photosystems from damage by increasing the energy dissipation (NPQ) (Tränkner et al., 2018). In addition, Mg can enhance photosynthetic N use efficiency (Wang J. et al., 2018), and like nitrogen, Mg affects light-harvesting through involvement in chlorophyll biosynthesis Kana and Govindjee, 2016.

Although the effect of nutrient deficiency on morphological traits is well described in the literature, for example, reduced leaf area, shoot growth, and shoot dry weight are among the most often described symptoms of K (Gerardeaux et al., 2010), P (Jacob and Lawlor, 1991), N (Mu et al., 2018), Mg (Guo et al., 2016), and Fe (Rout and Sahoo, 2015) deficiency, lower classification success obtained by morphological traits compared to multispectral and chlorophyll fluorescence traits could be explained by the fact that N, P, and K deficiency cause similar morphological changes. Namely, the highest misclassification of plants using morphological traits was found among these three groups. Similarly, Cadet and Samson (2011) obtained a significant reduction of leaf area under N, P, and K deficiency compared to control but did not find a significant difference in sunflower leaf area among these nutrient deficiency groups. However, recursive partitioning analysis identified total leaf area (TLA) and digital volume (DV) as the most important morphological traits for plant assignment into their respective nutrient deficiency groups from MT2 to MT4. For example, at MT2, TLA was used for the correct assignment of plants into the control group, whereas TLA and DV were used for plant assignment into the K group. At MT3, plants were assigned based on TLA into control, Fe, and K groups, whereas at MT4, DV was used for plants assignment into Fe and control, and DV and TLA were used for assignment of plants into P and Mg groups. These results indicate that recursive partitioning analysis can identify slight differences in the DV and TLA caused by different nutrient deficiencies and use it for the correct assignment of plants into their respective groups.

The better overall performance of linear discriminant analysis over recursive partitioning is in accordance with the results of Feldesman (2002) who showed that LDA was able to obtain slightly better correct classification than recursive partitioning for the set of morphometric variables of hominids. However, recursive partitioning results in a set of classification rules that use raw variables instead of canonical variates and are therefore easier to apply for the classification of new data and to interpret and relate to the underlying physiological processes.

This study has shown the usefulness of combining high-throughput phenotyping techniques with advanced data analysis to determine and differentiate nutrient deficiency stress. This study was conducted in a controlled environment with a controlled nutrient supply; thus, lower discrimination accuracy should be expected in the field, especially considering the possible co-occurrence of different nutrient deficiencies at the same time (Kalaji et al., 2018) or even co-occurrence of multiple abiotic and biotic stresses. However, similar analytical methods could be applied to develop discrimination models for other crop species, monocots, and dicots, although they show different nutrient uptake and use efficiency, as well as different patterns of nutrient deficiency symptoms. The classification accuracy of such models could be increased by increasing the number of plants in the study and using additional physiological criteria for discrimination among nutrient deficiency groups. For example, due to the differences in nutrient mobility within the plant, deficiency symptoms will occur on young leaves (less mobile nutrients) or old leaves (mobile nutrients) (Marschner, 1995), and by including this criterion in the analysis, better classification accuracy could be expected. Optimization of the classification in further experiments could provide a solid basis for developing specific sensor systems that could be used in the future to monitor the nutritional status of crops in the field. Since multispectral imaging is more accessible in agriculture than chlorophyll fluorescence imaging and because multispectral traits have shown the earliest response to nutrient deficiency, with high accuracy in plant assignment into their nutrient deficiency groups, these techniques/traits could be easier implemented in agriculture for nutrient deficiency detection/classification.

## Conclusions

Traits obtained using high-throughput phenotyping enabled high discrimination accuracy among studied nutrient deficiency groups. At early nutrient deficiency stress (MT1), highest classification success was achieved using multispectral traits and the lowest using morphological traits. In the subsequent measurements (MT2-MT4), chlorophyll fluorescence and multispectral traits achieved comparable classification success. While linear discriminant analysis achieved better overall classification accuracy, recursive partitioning was able to intrinsically identify variables within each group of traits and their threshold values that best separate the observations from different nutrient deficiency groups. The highest success in assigning plants into their respective groups was achieved based on selected multispectral traits. Selected chlorophyll fluorescence traits also showed high accuracy for the assignment of plants into control, Fe, Mg, and P deficit, but some misclassification occurred in the assignment of K and N deficit plants.

## Data availability statement

The raw data supporting the conclusions of this article will be made available by the authors, without undue reservation.

## Author contributions

BL and KC-S: conceptualization, resources, and funding acquisition. BL and TS: methodology, formal analysis, and visualization. TJ: investigation. TS: data curation. BL: writing—original draft preparation. TJ, KC-S, MŽ, DV, and TS: writing—review and editing. KC-S: project administration. All authors have read and agreed to the published version of the manuscript.

## Funding

This work was conducted as part of the CASEE-The ICA Regional Network for Central and South-Eastern Europe cooperation project Building the cooperation network in phenotyping toward crop optimization and as part of the project KK.01.1.1.01.0005 Biodiversity and Molecular Plant Breeding, Centre of Excellence. Funds received from CASEE, Ref No: CASEE fund 2021-1 was used for the open access publication fee.

## Conflict of interest

The authors declare that the research was conducted in the absence of any commercial or financial relationships that could be construed as a potential conflict of interest.

## Publisher's note

All claims expressed in this article are solely those of the authors and do not necessarily represent those of their affiliated organizations, or those of the publisher, the editors and the reviewers. Any product that may be evaluated in this article, or claim that may be made by its manufacturer, is not guaranteed or endorsed by the publisher.
